# CIMBL55: a repository for maize drought resistance alleles

**DOI:** 10.1007/s44154-023-00091-4

**Published:** 2023-05-19

**Authors:** Tian Tian, Feng Qin

**Affiliations:** State Key Laboratory of Plant Environmental Resilience, College of Biological Sciences, China Agricultural University, Beijing, 100193 China

**Keywords:** Maize, Drought resistance, Genome assembly, Genetic variants

## Abstract

Droughts threaten crop yields worldwide. Compared to other major staple cereal crops, maize (*Zea mays*) is especially sensitive to drought, which can cause dramatic fluctuations in its yield potential. Natural maize populations contain many superior alleles that can enhance drought resistance through complex regulatory mechanisms. We recently de novo assembled the genome of a prominent drought-resistant maize germplasm, CIMBL55, and systematically dissected the genetic basis for its drought resistance on the genome, transcriptome, and epigenome levels. These analyses revealed 65 favorable drought resistance alleles in CIMBL55. Subsequently, we genetically verified the functions of the drought resistance genes *ZmABF4*, *ZmNAC075*, and *ZmRtn16* and unraveled the function of *ZmRtn16* on a molecular level.

This article highlights our recent study (Tian et al. 2023), in which we assembled the genome of a prominent drought-resistant maize (*Zea mays*) germplasm, CIMBL55, and identified several superior drought resistance alleles. Crops are subjected to numerous environmental stresses throughout their life cycle (Hirt et al. 2023). Maize, as one of the most widely cultivated crops worldwide, suffers severe or even total production losses due to drought stress. Maize originated in Mexico ~ 9,000 years ago, and as new varieties were selected, its cultivation region expanded from low to high latitudes (Liang et al. 2021). Interestingly, varieties with a tropical or subtropical pedigree tend to have higher drought resistance than varieties from temperate areas. Drought resistance is a quantitative trait with a complex genetic basis. Population genetics studies have helped identify superior drought resistance alleles and uncover the molecular mechanisms of drought resistance, contributing to the breeding of drought-resistant maize varieties with enhanced yield potential. A genome-wide association study of maize seedling survival rate after drought treatment, using 368 lines of an association panel, identified 83 genetic variants surrounding 42 candidate genes that explained approximately 55.2% of the phenotypic variation (Wang et al. 2016). In a subsequent study, expression quantitative trait locus (eQTL) analysis of seedlings exposed to well-watered, moderate drought (70% relative leaf water content), and severe drought (58% relative leaf water content) conditions identified 19,566 (26.6%) static and 54,007 (73.4%) dynamic eQTLs, encompassing 97 genes associated with drought resistance (Liu et al. 2020). Later studies identified superior alleles of several candidate genes, such as *ZmVPP1*, *ZmNAC111*, *ZmTIP1*, *ZmSRO1*, and *ZmABH2*, in the maize inbred line CIMBL55, which directly or indirectly function in the regulation of water uptake and water loss to maintain viability under water deficit (Rodrigues et al. 2019; Yang and Qin 2023). However, many other drought resistance loci remain to be characterized in maize.

Third-generation sequencing technologies have produced high-quality genome assemblies for maize and other plants with complex genomes. The 5th edition of the maize reference genome, B73 has fewer gaps than previous versions, and has a contig N50 greater than 50 Mb (Hufford et al. 2021). Since the release of the B73 reference genome, dozens of additional genome assemblies have been released, including Mo17 (Sun et al. 2018), small kernel (SK) (Yang et al. 2019), A188 (Lin et al. 2021), and K0326Y (Li et al. 2020), along with 26 nested association population founders (Hufford et al. 2021), 7 wild relatives of maize (Chen et al. 2022), and 12 maize germplasms from different heterotic groups (Wang et al. 2023). These assemblies have enhanced genomics-based breeding and the analysis of adaptive evolution. For example, the genome for maize inbred line SK, which has small kernels and low productivity, was assembled to analyze yield traits. An 8.9-kb insertion upstream of *ZmBAM1d* was identified in the B73 genome that promotes its expression and increases hundred-kernel weight, possibly due to altered chromatin interactions and methylation levels (Yang et al. 2019). Furthermore, the assembly A188 revealed that a copy number variation in *carotenoid cleavage dioxygenase 1* (*ccd1*) between A188 and B73 alters carotenoid accumulation and results in different seed colors (Lin et al. 2021). Finally, Huang et al. used a high-quality teosinte haplotype assembly to identify a superior allele of *TEOSINTE HIGH PROTEIN 9* (*THP9*) in the wild ancestor of maize that confers increased seed protein content (Huang et al. 2022).

These high-quality genomes counteract the bias inherent in using a single reference genome and provide an extensive allele resource repository. However, these genomes do not include any germplasm noted for drought resistance. The maize inbred line CIMBL55 has a tropical and subtropical pedigree and is a well-known drought-resistant germplasm (Wang et al. 2016). Using 160 × PacBio single-molecule real-time sequencing, 160 × BioNano single-molecule optical mapping, and 35 × Hi-C sequencing technologies, along with state-of-the-art assembly strategies and annotation pipelines, we generated a contiguous CIMBL55 genome assembly containing 38,439 protein-coding genes and 83.95% repeats (Tian et al. 2023). We identified large structural variations between CIMBL55 and 30 other high-quality assembled maize genomes, including the reference genome B73; these variations may contribute to the drought resistance of this unique germplasm.

We analyzed gene synteny, structural variations, and epigenetic differences. First, we compared the gene synteny maps of CIMBL55 and B73 or Mo17 and found differences in gene order and gene presence/absence. Genes within syntenic blocks on homologous chromosomes tended to share a common order, while non-syntenic regions had undergone massive changes, such as chromosome rearrangements and transposon hopping (Wang et al. 2012). We found a special class of genes named Class2 (29% of total maize genes), which are syntenic as well as duplicated and include a higher percentage of abscisic acid signaling and stress response genes compared to other gene classes. This phenomenon is consistent with the trend of evolutionary conservation of key signaling factors and hub genes (Madan Babu and Teichmann 2003). Next, we used four state-of-the-art strategies, including assembled contig-based (MUMmer + SyRi and blasr + smartie-sv) and clean read-based (pbmm + pbsv and ngmlr + sniffle) strategies, to identify DNA sequence variations, including single nucleotide variations, small insertions/deletions (indels), large insertions, large deletions, inversions, translocations, and duplications between CIMBL55 and the 30 other maize genome assemblies. We obtained some overlaps from different strategies and programs, highlighting the importance of combining multi-layer algorithms to identify genomic variation in complex genomes. In total, we identified 127,742,577 single nucleotide polymorphisms (SNPs), 14,499,458 indels, and 3,081,556 structural variants (SVs) on a pan-genome level.

Next, we developed a bi-directional SV identification strategy to accurately identify SVs between two genomes, which is especially important for predicting their potential regulatory roles. Insertion variations within the flanking region of genes may introduce or disrupt *cis*-acting elements or change the chromatin status. Furthermore, a translocation within a coding region may abolish gene function. To investigate epigenomic differences in conserved and variable regions, we conducted whole-genome bisulfite sequencing of three maize inbred lines (CIMBL55, B73, and Mo17). In conserved regions, we found many regions that were hypomethylated (11,019) and hypermethylated (12,066) in CIMBL55 compared to B73 or Mo17 and identified 7,170 genes near those differentially methylated regions. However, these genes showed no significant differences in gene expression or DNA methylation among the three inbred lines. Interestingly, we found that DNA methylation variations were more common in variable regions than in conserved regions. About 80% of inserted sequences were located in regions containing at least one type of DNA methylation. We found that clusters of inserted sequences with higher CHH (where “H” is A, C, or T) methylation levels were enriched in DNA transposon of Harbinger (DTH), which may impact the expression of proximal genes. Some members of the NAC (NAM, ATAF, and CUC) family of transcription factors function as positive regulatory factors of drought resistance, and their overexpression leads to increased yield under water deficit conditions (Al Abdallat et al. 2014). Two insertional sequences upstream of *ZmNAC075*, S-9041 (182 bp) and S-1425 (5,123 bp), were hypermethylated in B73, but not in CIMBL55, significantly reducing *ZmNAC075* expression in B73. Furthermore, when the CHH methylation level at these two insertional sequences in B73 was diminished by knocking out *Zmdrd1*, which functions in RNA-directed de novo DNA methylation, the expression of *ZmNAC075* was enhanced. These results suggested that hypermethylation of the two inserted sequences in B73 inhibits *ZmNAC075* expression and compromises drought resistance.

We identified 208,036 B73 reference-based and 336,817 CIMBL55 reference-based SVs and genotyped them in an association panel consisting of 368 inbred lines. Analysis of the association of these newly identified SVs with drought resistance provided a comprehensive and accurate genetic dissection for the trait. Several newly identified SVs showed strong linkage disequilibrium with SNPs previously significantly associated with drought resistance, suggesting that low-density SNPs can be used to pinpoint the potential causative region, and high-density variations, especially SVs, are beneficial to unravel the causal variants for gene expression regulation. For example, Zhang et al. established that *ZmRtn16* encodes a reticulon-like protein that is associated with the endoplasmic reticulum (ER) and functions in protein trafficking from the ER to the Golgi apparatus as part of autophagic flux in endosperm aleurone cells during seed germination (Zhang et al. 2020). In this research, ZmRtn16 interacts with the A and E3 subunits of the tonoplast proton pump (the vacuolar H^+^-ATPase) and plays an essential role in directing the two subunits to the tonoplast. Loss of function of *ZmRtn16* resulted in reduced vacuolar H^+^-ATPase activity and compromised drought resistance. Our study identified a deletion, S-2290 (28 bp), in the 3′ untranslated region of *ZmRtn16*^*CIMBL55*^ compared with *ZmRtn16*^*B73*^; this deletion strongly enhanced its expression and is in strong linkage disequilibrium with a previously identified SNP associated with drought resistance. Removal of this 28-bp fragment from the *ZmRtn16*^*B73*^ allele dramatically increased its transcript abundance to a level similar to that of the *ZmRtn16*^*CIMBL55*^ allele. An RNA binding motif within the 28-bp fragment was predicted based on a search of the online CISBP-RNA (Catalog of Inferred Sequence Binding Proteins of RNA) database (http://cisbp-rna.ccbr.utoronto.ca), which contains a collection of experimentally identified and inferred RNA binding motifs. The increased expression of *ZmRtn16 *^*CIMBL55*^ relative to *ZmRtn16*^*B73*^ may be mediated by RNA binding proteins at the post-transcriptional level.

High-quality genome assemblies enable accurate and efficient identification of key components of genomes, such as exact gene-coding sequences, transcriptional regulatory elements, transcript isoforms, non-coding RNAs, and transposon structures. Efficient and accurate identification of complex structural variations and prediction of their associations or contributions to interesting traits demand further innovations in algorithms and their integration with deep neural network frameworks of artificial intelligence. Furthermore, high-throughput phenotyping technology for temporal and spatial developmental data are necessary for mining superior drought resistance alleles from the huge genetic resource of maize. Finally, exploiting these drought resistance alleles via gene editing will help to develop robust crops that are more resilient to the stresses caused by global climate change.

## Fundings

This research was supported by Beijing Outstanding Young Scientist Program (BJJWZYJH01201910019026) and Chinese Postdoctoral Science Foundation (2019M660874, 2021T140714).

## Data Availability

Not applicable.
